# District-led malaria surveillance and response as an effective way to manage malaria upsurges following the withdrawal of indoor residual spraying: a case study from Nwoya District, northern Uganda

**DOI:** 10.1186/s12936-022-04066-0

**Published:** 2022-02-19

**Authors:** Anthony Nuwa, Janet Oola, Sam Okot Obonyo, Mitra Feldman, Shirah Karungi, Edmound Kertho, David Salandini Odong, Isaac Kimera, Godfrey Magumba, Geofrey Beinomugisha, Alexandra Chitty, James Tibenderana, Jimmy Opigo, Francis Abwaimo

**Affiliations:** 1 Malaria Consortium Uganda Country Office, Kampala, Uganda; 2 Nwoya District Local Government, Nwoya, Uganda; 3 Independent Public Health Consultant, Monteverde, Costa Rica; 4 Malaria Consortium, Green House, 224-254 Cambridge Heath Road, E2 9DA , London, UK; 5 National Malaria Control Division, Ministry of Health, Kampala, Uganda

**Keywords:** Malaria surveillance and response, Malaria surveillance and response team, Indoor residual spraying, Malaria positivity rates

## Abstract

**Background:**

Malaria remains the number one cause of morbidity and mortality in Uganda. In 2009, the United States President’s Malaria Initiative (PMI) funded an indoor residual spraying (IRS) project in 10 mid-northern districts, resulting in marked reductions in malaria prevalence over 5 years, from 62.5 percent to 7.2 percent. When the project ended and IRS withdrawn, malaria prevalence increased exponentially to pre-IRS level of 63 percent in 2016 and was characterized by frequent life-threatening upsurges that were exacerbated by a weak national led malaria surveillance system with delayed and piece meal responses. Malaria Consortium, in collaboration with Nwoya district local government implemented a district led malaria surveillance and response system. This study was conducted to compare the impact of District led and national led surveillance and response systems on overall malaria burden in two sub-counties in Nwoya district, Northern Uganda.

**Methods:**

The assessment was conducted between week 41 of 2018 and week 10 of 2019 in Anaka and Alero sub counties following the shift from the national to district led malaria surveillance and response system. A district multi-sectoral malaria response taskforce team, known as the District Malaria Surveillance and Response Team (DMSRT), was formed by the Nwoya District Health Team (DHT). The DMSRT was trained and equipped with new surveillance tools for early detection of and response to malaria upsurges within the district, and were mandated to develop a costed district specific malaria response plan.

**Results:**

All (18) targeted health facilities provided weekly malaria reports and continuously updated the malaria normal channel graphs. There was an overall reduction in weekly new malaria cases from 12.9 in week 41 of 2018 to 6.2 cases in week 10 of 2019. Malaria positivity rates (TPR) for Alero and Anaka sub-counties reduced from 76.0 percent and 69.3 percent at week 42 of 2018 to 28 percent and 30.3 percent, respectively at week 10 of 2019.

**Conclusions:**

Malaria surveillance and response, with precisely targeted multipronged activities, when led and implemented by local district health authorities is an effective, efficient, and sustainable approach to prevent malaria upsurges and associated morbidity and mortality.

## Background

Malaria remains the number one cause of morbidity and mortality in Uganda [1]. The World Health Organization (WHO) lists weak surveillance systems as one of the key challenges that prolong the fight against malaria in various countries, because they compromise the ability to track gaps in programme coverage and changes in disease burden [2]. Thus, the WHO emphasizes the use of high-quality surveillance data for decision-making to drive tailored responses consistent with national and subnational malaria control goals. In 2009, the United States President’s Malaria Initiative (PMI) supported the implementation of an indoor residual spraying (IRS) programme in 10 districts in northern Uganda with high levels of malaria transmission intensity, including Nwoya District [3, 4]. The programme achieved coverage levels consistently above 95 percent and resulted in marked reductions in the malaria burden, and a prevalence of just seven percent [5, 6] down from 62.5 percent.

The IRS programme was suspended in the 10 northern districts at the end of 2014, with hopes that the gains achieved would be sustained following the 2014/2015 universal coverage campaign (UCC) with long-lasting insecticidal nets (LLINs). Unfortunately, although planned to coincide with the UCC, the discontinuation of IRS began before the LLIN distribution was completed. Existing interventions were not sufficient to maintain the reduced malaria transmission intensity which was achieved with IRS. The ecosystem regained its malariogenic potential and 4–18 months after discontinuation of IRS, the TPR values among outpatients aged < 5 years increased by an average of 3.29 percent per month, returning to baseline levels of 60–80 percent [7]. During this time, malaria transmission in some districts, such as Nwoya, was characterized by frequent periodic upsurges that persisted for over 3 years [6]. These upsurges were exacerbated by a weak national led malaria surveillance and response system, which only relied on monthly malaria reports captured in the District’s Health Information System (DHIS2).

**Fig. 1:**
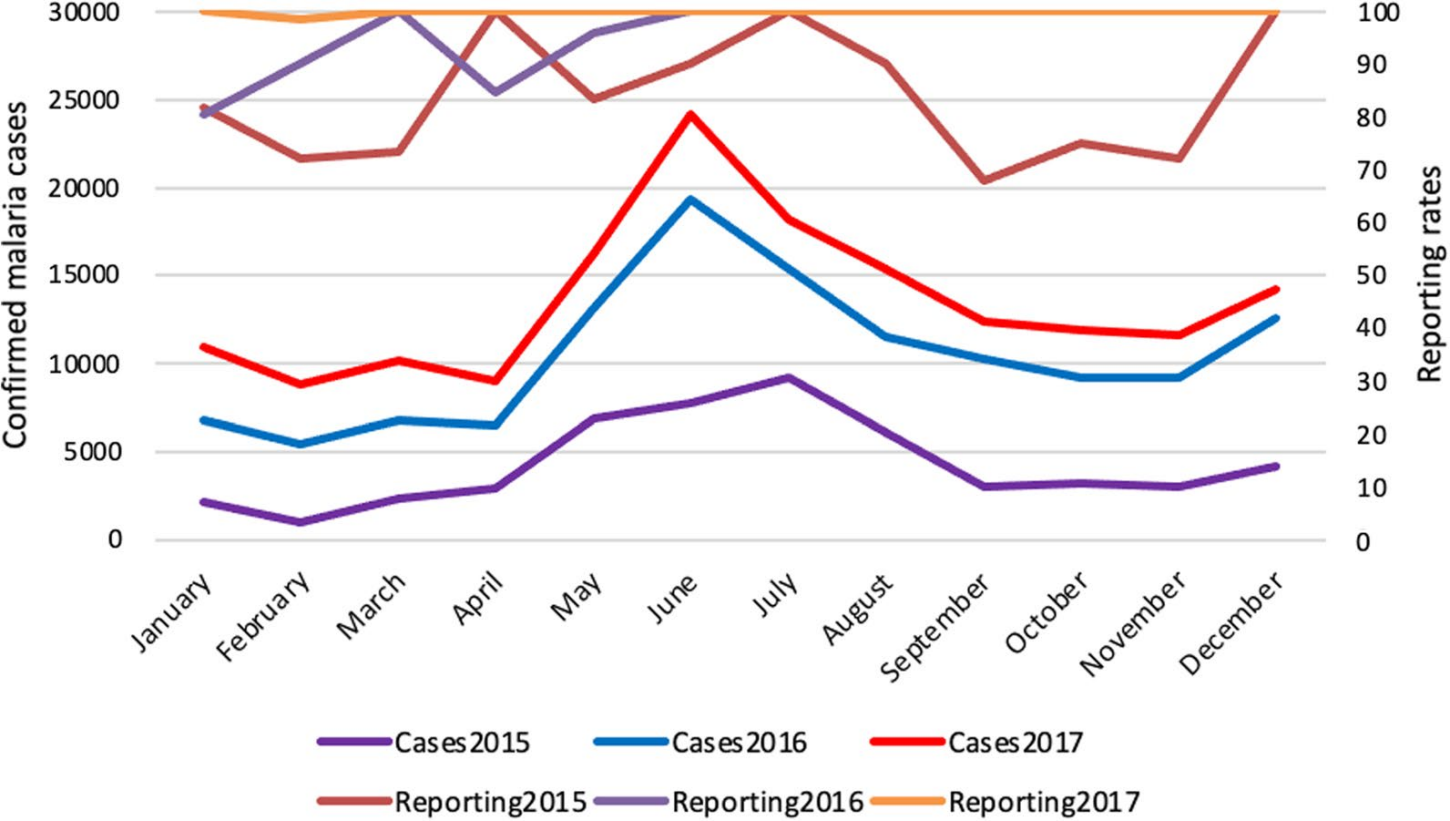
The monthly malaria surveillance report for Nwoya district for 2015–2017 (Source DHI2 accessed on 08/03/2021)

The national led malaria surveillance and response system was passive and suffered from a lack of surveillance coverage in remote communities, lack of integration of data from other sources beyond government health facilities, such as the private sector and community health workers (known as Village Health Teams, VHTs), inadequate health information architecture to capture complete and timely routine health data, as well as reporting of aggregated district level instead of stratified sub-district malaria data. VHTs in Uganda treat many children under 5 years old through integrated community case management (iCCM), yet the malaria cases they attended to were never captured in this surveillance system as the majority of VHTs were not reporting into the DHIS 2. Any increase in malaria cases was only noticed at the end of each month, after the districts had submitted their overall monthly reports, as demonstrated in Fig. 1. Therefore, any national led response was often late, misaligned or mistimed and carried out with lack of district-level ownership. This system was contrary to the guidelines in the WHO Global Technical Strategy for Malaria 2016–2030 (GTS), which recommends the transformation of malaria surveillance into a core malaria intervention to allow for active identification, tracking, classification and response for all malaria cases to effectively support case management [2, 8]. The GTS recommendation also emphasizes the provision of parasite-based diagnosis and treatment at health facility and in the community through community health workers. Uganda adopted the WHO recommendation of co-opting surveillance as a core malaria intervention in 2019.

In last quarter of 2018, the UK DFID (Now the UK Foreign, Commonwealth and Development Office) funded Strengthening Uganda’s Response to Malaria (SURMa) project commenced its operations in these post-IRS districts and supported the shift from the national-led monthly surveillance to a district led, weekly malaria surveillance and response system. This paradigm shift increased the focus on training district malaria programme staff on surveillance, epidemic preparedness, and response. A three-stage comprehensive response plan was developed for the affected districts, among which a key intervention was to set up District Malaria Surveillance and Response Teams (DMRTs). This study compares the impact of the national led system with the district led surveillance and response systems on overall malaria burden in Nwoya District in Northern Uganda.

**Fig. 2:**
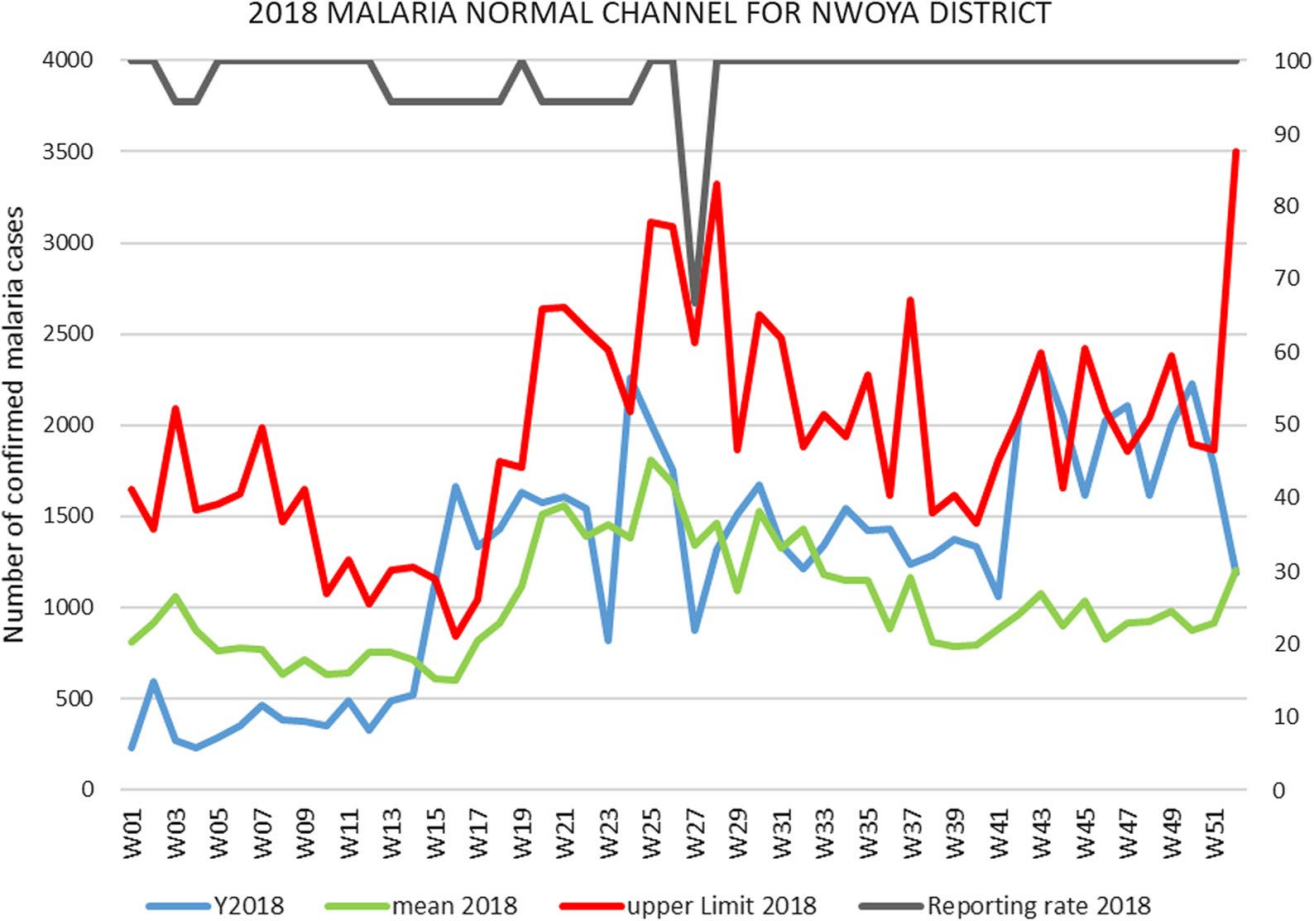
Malaria Normal Channel graph, Nwoya District 2018 (Source: DHIS2)

Figure 2 is a demonstration of the weekly district led surveillance system. It shows the district level malaria normal channel graph, which the District Health Team (DHT) used to monitor local malaria cases. The normal channel graphs are plotted every week at both the district and health facility levels to enable the District surveillance focal persons and health facility surveillance focal persons to detect and respond in a timely way to any malaria upsurge in the district or a specific catchment area. As shown in the figure, there was a sudden increase in the number of malaria cases in week 41 of 2018, which prompted the district to conduct further analysis of sub county level malaria data. Unlike with the 2016 upsurge, which was brought to the attention of the Ministry of Health (MoH) by the Ugandan press, the 2018 malaria upsurge was detected by the DHT, following the strengthened surveillance and response systems.

## Literature review

The concept of surveillance and response evolved from the original vision of the general and open-ended-term ‘surveillance’, defined as “the continuous and systematic collection, analysis and interpretation of disease-specific data, and the use of that data in the planning, implementation, and evaluation of public health practice” [8, 9]. Timely dissemination of surveillance results can improve planning, implementation, and evaluation of interventions [8].

Surveillance is, therefore, the basis of operational activities in settings of any level of transmission. Its objective is to support reduction of the burden of malaria, eliminate the disease and prevent its re-establishment [10]. In high transmission settings, malaria surveillance data is used to monitor trends in the number of cases and deaths, over time and by geography; the characteristics of people infected or dying from malaria; and the seasonality of transmission. In such settings, surveillance data can also be used to stratify geographical units by their malaria prevalence or annual parasite incidence, to better target interventions and optimize resource allocation. By strengthening capacity to assess trends and respond without delay, surveillance itself can become an intervention [10]. Surveillance is the third pillar in the WHO GTS for malaria and is earmarked as a key intervention for malaria control, including improved monitoring and evaluation as well as stratification of geographical areas by burden [2]. The Global Malaria Programme (GMP) has embarked on an intensified process of improving national surveillance systems and the use of data for programmatic decision-making [11]. Surveillance systems should be robust enough to detect and respond to malaria upsurges within 2 weeks of onset. To be truly effective, strong national surveillance systems with malaria upsurge early warning triggers, need to be linked to efficient and high quality, localized surveillance systems [12].

Surveillance and response systems provide an early warning function and serve to provide data that can be used to detect and respond to outbreaks or public health threats, such as malaria upsurges, in a timely and appropriate manner [8, 9, 12]. The strength of such systems should be judged based on:

- Early detection of case load by the system.
- Epidemiological investigation undertaken.
- Timely initiation of response and
- use of the surveillance data to guide the public health response.

The main objective of this study was to assess the district led and the national led, malaria surveillance and response systems in terms of effectiveness and sustainability to prevent malaria upsurges and associated morbidity and mortality.

## Methods

In 2018, the SURMa project, in collaboration with the Uganda National Malaria Control Division (NMCD) and the Nwoya DHT, revised the malaria surveillance system and set up a district-led surveillance system. An independent DMSRT was created to manage and implement this surveillance system. The DMSRT was comprised of technical personnel, according to Uganda’s Guidelines for Preparedness and Response for Malaria Epidemics [13] e.g., environmental health officer, clinician, laboratory technician, Nurses, biostatistician, health educator, a logistician, and public, private and community health workers.

**Fig. 3:**
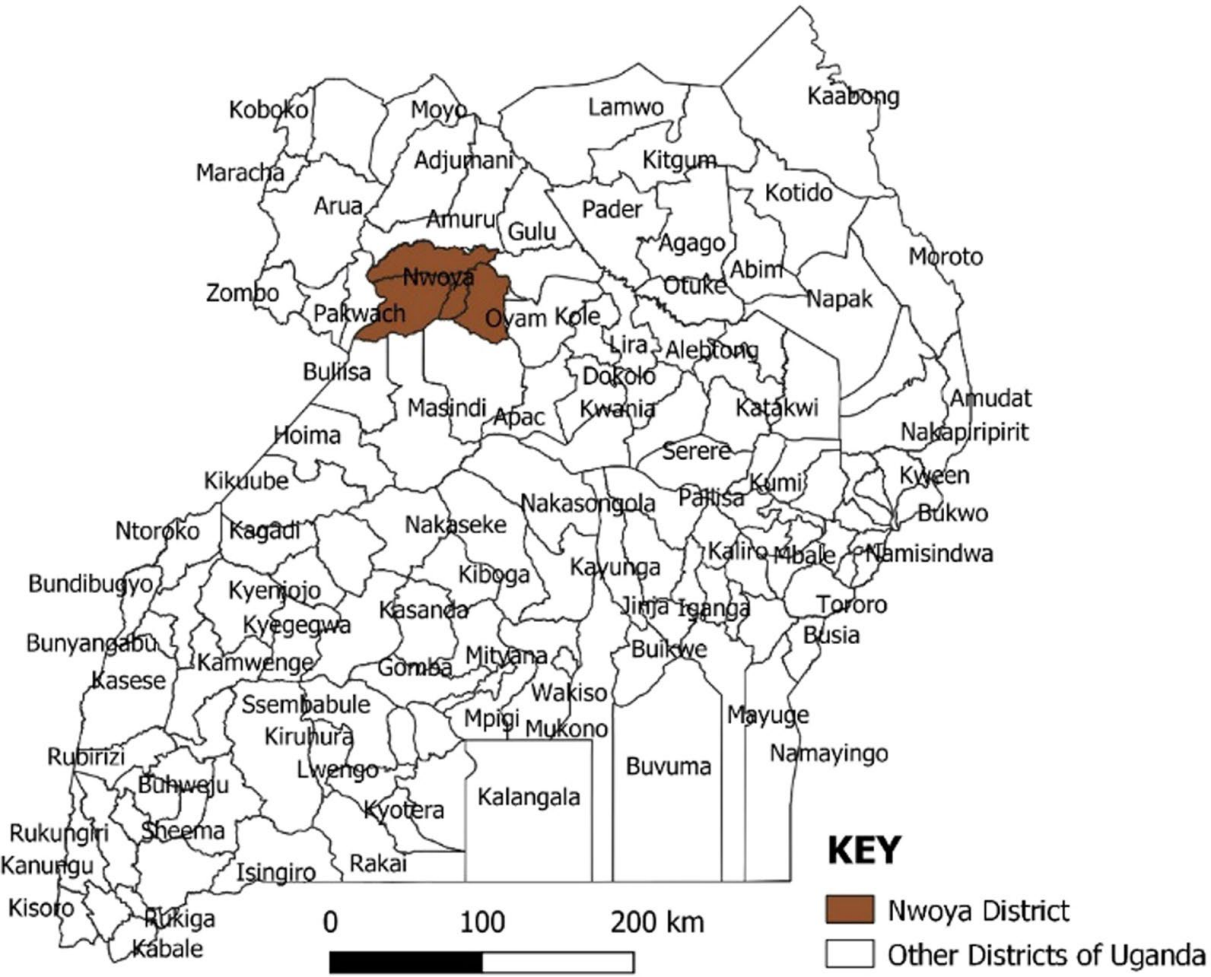
Nwoya District and most affected sub-counties

The DMSRT conducted a rapid assessment using the DHIS 2 to map out specific geographic areas most affected by malaria and as result the two sub-counties namely Alero and Anaka (Fig. 3), where this study was conducted were identified. In addition, a multi-sectoral management taskforce was also formed which includes key district sector leads from health, education, engineering, and community development, as well as implementing partners to oversee and support the DMSRT. The SURMa project supported the training of the DMSRT, equipped all health facilities (public and private) with malaria normal channel graphs and supplied reporting tools to the VHTs.

Health Facility (HF) record reviews and mapping exercises were conducted and the specific villages within these sub-counties most impacted were identified for targeted interventions. The mapping exercise involved, using the HF data on malaria cases to identify the villages within the catchment area of the HFs with the highest malaria cases. This was followed by conducting micro-census to determine the total population as well as the number of vulnerable groups, such as children under 5 years and pregnant women, residing in these village; identifying the available community resources such as VHTs and local leaders to mobilize people for the test and treat campaigns. A field visit was carried out to the respective sub-counties to assess the awareness of the outbreak among the community and the availability of artemisinin-based combination therapy (ACT) and rapid diagnostic tests (RDTs) at the HFs and VHTs. The Nwoya district vector control officer did preliminary entomological surveillance, which was comprised of mosquito collection using spray catches and CDC light traps, in some villages and noted increased mosquito densities. A planning meeting was convened by the DHT to inform all relevant stakeholders of the rapid assessment findings, mobilize resources and develop a comprehensive district response plan, based on the findings and guided by policy.

Key interventions, categorized as immediate, intermediate and long-term were designed, agreed on by key stakeholders and approved by the MoH. The expected outcome of the immediate intervention was to avert deaths through the testing and treatment of all cases; the intermediate objective was to bring down the upsurge, while the long-term goal was to sustain the gains achieved.

Using the CDC field epidemiological training manual, all, in-charges and the staff in charge of records at health facilities from the two sub-counties were (both public and private) were trained and provided with malaria surveillance tools, such as weekly malaria normal channel graphs. The remaining health workers from these facilities and VHTs were trained and provided with tools and facilitation to conduct regular targeted Test, Treat and Track outreach activities in the most affected areas as part of surveillance response system. All VHTs in Alero and Anaka sub-counties were trained on malaria epidemic response and provide with reporting tools and more mRTs to conduct testing during home visits in their villages as part of malaria surveillance response. During home visits and test and treat outreaches, VHTs and health workers would also identify pregnant women and mobilize them to attend antenatal care (ANC) and receive intermittent preventive treatment in pregnancy (IPTp) for malaria.

The HFs conducted test (using RDTs), treat and track outreach activities, as per the national malaria control plan, in areas where out-patient department (OPD) records indicated high malaria cases. Of the 7634 persons tested during these outreaches, 5355 (70 percent) were positive for malaria and were given ACTs, as per the national guidelines. Malaria outreach activities were also integrated into other existing healthcare activities such as the Expanded Program of Immunization (EPI), HIV/AIDS outreach, etc. There was no reactive case detection component to the response, however, a total of 10 test and treat outreach activities were conducted in catchment areas/villages from five health facilities with high malaria caseloads. These outreach activities were conducted by staff from the public HFs, with support from the various implementing partners operating in Nwoya District.

To ensure and sustain adequate stock of key malaria commodities [ACT, RDTs, sulfadoxine-pyrimethamine (SP) for IPTp] at the facilities and for the VHTs in the targeted sub-counties, regular monthly stock taking were conducted by the DMSRT in case of pending stock outs, key actions were made. These actions included the redistribution of commodities from health facilities to the VHTs for iCCM as well as from the neighbouring districts that had overstocks of these commodities to replenish the HF stock. This was done as part of surveillance response to ensure no stock out of commodities and was primarily conducted in the two sub-counties (Alero and Anaka).

Social behaviour change communication (SBCC) played a key role in the response against the malaria upsurge in Nwoya district. More than 3,400 SBCC materials were distributed through places of worship, schools, health facilities, community dialogue meetings, and VHTs. These materials were comprised of 22 key family health practice talking points, on a two-page flyer. Additionally, a total of 12 weekly radio talk shows and over 200 radio announcements/jingles were conducted during the response. The messages focused on the importance of consistent use of LLINs and seeking immediate medical attention in case one felt feverish or unwell. The HFs also conducted a total of eight malaria outreach activities during community sensitizations that focused on mass testing and treatment of malaria positive cases and promotion of malaria prevention and control best practices.

### Ethical considerations

This assessment was focused on electronic data in DHIS2. The investigation was determined to be non-human subject research according to Uganda’s research guidelines [14]. Authorization of the study was obtained from the District Health Officer (DHO). The Health facility staff who participated in data use in preparation of the malaria normal channel graphs were provided with information that their participation was voluntary and the activity was meant to build their capacity in malaria epidemic detection and their refusal would not lead to any consequences.

## Results and discussion

**Fig. 4:**
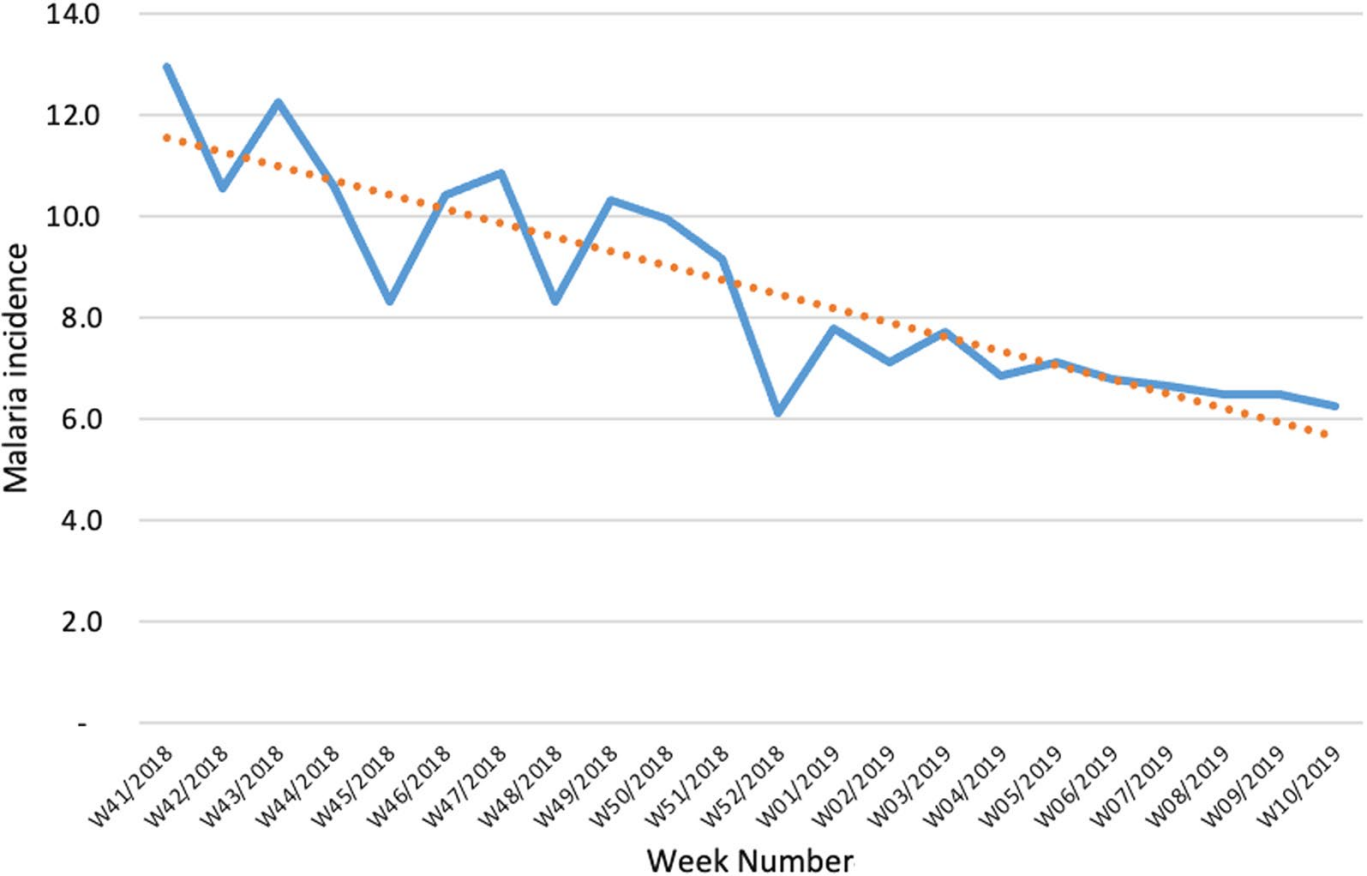
Trends of malaria report new cases for Nwoya district from week 41 of 2018 to week 10 of 2019 (Source: DHIS2)

Overall, Nwoya district registered a general reduction in malaria incidence reported at HFs from 12.9 cases per week in week 41 of 2018 to 6.2 cases per week in week 10 of 2019 (see Fig. 4). These two-time points were during the rainy and high malaria transmission seasons i.e., October and March.

**Fig. 5:**
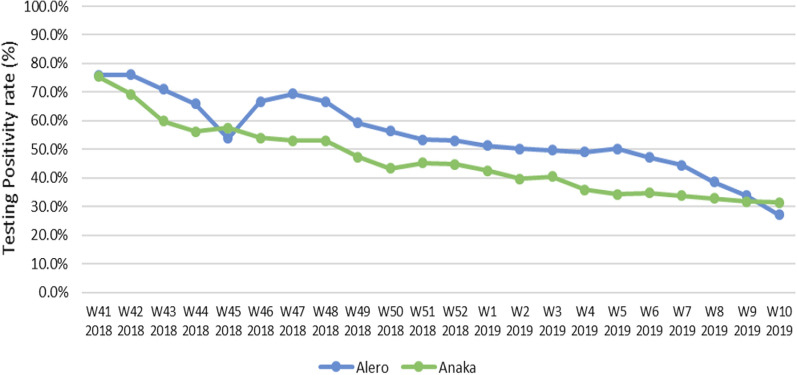
Trends of Total Malaria Positivity Rates for Alero and Anaka sub-counties from week 42 of 2018 to week 10 of 2019

**Fig. 6:**
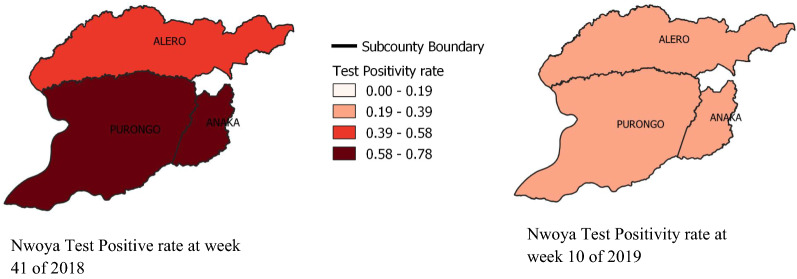
Geospatial maps showing Malaria situation in the study sub-counties before and after the implementation of upsurge response plan activities

As demonstrated in Fig. 5, the Malaria Positivity Rates (TPR) for Alero and Anaka sub-counties reduced from 76.0 percent and 69.3 percent at the beginning of the intervention (week 41/2018) to 28 percent and 30.3 percent, respectively, after the intervention at week 10 of 2019. Figure 6 is a geospatial visual representation of the changes in malaria positivity rates before and after implementation of malaria upsurge response in the two sub-counties.

The study, depicted that improvements as result of shifting from national to district led surveillance and response system enabled Nwoya district to effectively detect and respond to malaria upsurges with minimum external support. Improvements in data use by medical and non-medical staff at both the district and health facility levels, and weekly use and updating of malaria normal channel graphs using their own records, meant the district could conduct targeted outreach activities in villages identified as having high malaria incidence. In this way, the district was able to effectively raise awareness among partners and the general public, including the media, and respond in a timely manner.

The routine VHT follow-up and mentorship led to improved community-based malaria reporting from 78 percent during the October–December 2018 quarter to 100 percent during the January–March 2019 quarter. The quality of malaria data greatly improved. For instance, Nwoya district used to report treatment of an average of 23% of the negative cases before the intervention. But by week 10 of 2019, this percentage had significantly reduced to less than 5% as a result of sustained mentorships on data quality improvements in all health facilities. Lastly, according to DHIS2 data, there was improved adherence to malaria RDT test results by health facilities in Nwoya District, as indicated by reduced dispensing of ACT to RDT negative patients from an average of 34.5 percent in 2017, to 23 percent in 2018 and 2.5 percent in week 10 of 2019.

## Conclusions

This study demonstrates that shifting from a national-led to district-led malaria surveillance and response system in Nwoya District resulted in the early and timely detection and response to malaria upsurges. This study also illustrates that district-led malaria surveillance and response, coupled with targeted multipronged activities, is a feasible approach to avert high malaria morbidity and mortality in Uganda.

### Recommendations

The Ugandan MoH, through its NMCD, should support districts with high malaria endemicity and that are prone to recurrent upsurges, to take the lead in malaria surveillance and response activities. This study demonstrates that district-led surveillance and response is more feasible, impactful and sustainable.

All high malaria endemic districts that are prone to frequent life-threatening malaria upsurges should set up a task force with a technical DMSRT for the early detection and timely response to upsurges. The DMSRT should regularly and systematically review both epidemiological and entomological data to detect early malaria upsurge warnings.

The locally implemented multipronged targeted activities such as test, treat and track outreach activities in high malaria endemic villages, SBCC and use of malaria normal channel graphs at health facility levels should be prioritized and provided with enough resources.

### Limitations of the study

The malaria normal channel graphs were drawn using data from health facilities and does not include data from the community level where VHTs treat children with malaria through the iCCM program. This means that the trends in the malaria normal channel graphs  under-represent the occurrence of malaria in the population. Data quality at some health facilities was not always reliable: characterized with incomplete or late, and sometimes inaccurate, submissions.

## Data Availability

The datasets used and/or analysed during the current study are available from the corresponding author on reasonable request.

## Abbreviations

ACT: Artemisinin-based combination therapy
ANC: Antenatal care
DFID: Department for International Development
DHIS: District Health Information System
DHT: District Health Team
EPI: Expanded Program of Immunization
GMP: Global Malaria Programme
HFs: Health facilities
ICCM: Integrated community case management
IPTp: Intermittent preventative treatment in pregnancy
IRS: Indoor residual spraying
LLINs: Long lasting insecticidal nets
MoH: Ministry of Health
NMCP: National malaria control programme
NMS: National medical stores
OPD: Outpatient Department
PMI: President’s Malaria Initiative
RDT: Rapid diagnostic test
DMSRT: District Malaria Surveillance and Response Team
SBCC: Social behaviour change communication
SP: Sulfadoxine-pyrimethamine
SURMa: Strengthening Uganda’s Response to Malaria
UCC: Universal coverage campaign
VHT: Village health team
WHO: World Health Organization
